# Supplementary material for: Modeling glioblastoma heterogeneity as a dynamic network of cell states
Source: Mol Syst Biol. 2021 Sep 16;17(9):e10105. doi: 10.15252/msb.202010105 (PMC8444284; doi:10.15252/msb.202010105)
Supplement: Supplementary file 5 — Source Data for Figure 3 [file MSB-17-e10105-s001.zip › Figure3A_sourcedata/GSEA_3065/hallmarks_state3.GseaPreranked.1623416274744/HALLMARK_DNA_REPAIR.html]

Details for gene set HALLMARK\_DNA\_REPAIR[GSEA]

|  || Dataset | state3 |
| Phenotype | NoPhenotypeAvailable |
| Upregulated in class | na\_neg |
| GeneSet | HALLMARK\_DNA\_REPAIR |
| Enrichment Score (ES) | -0.5161851 |
| Normalized Enrichment Score (NES) | -1.9139694 |
| Nominal p-value | 0.0 |
| FDR q-value | 1.7777778E-4 |
| FWER p-Value | 0.001 |
Table: GSEA Results Summary

  

Fig 1: Enrichment plot: HALLMARK\_DNA\_REPAIR      
 Profile of the Running ES Score & Positions of GeneSet Members on the Rank Ordered List

  

| PROBE | GENE SYMBOL | GENE\_TITLE | RANK IN GENE LIST | RANK METRIC SCORE | RUNNING ES | CORE ENRICHMENT || 1 | DAD1 |  |  | 144 | 0.313 | 0.0029 | No |
| 2 | SEC61A1 |  |  | 193 | 0.290 | 0.0155 | No |
| 3 | ARL6IP1 |  |  | 210 | 0.278 | 0.0310 | No |
| 4 | NUDT9 |  |  | 400 | 0.219 | 0.0228 | No |
| 5 | POLR2A |  |  | 472 | 0.204 | 0.0273 | No |
| 6 | REV3L |  |  | 506 | 0.198 | 0.0358 | No |
| 7 | DGCR8 |  |  | 577 | 0.189 | 0.0395 | No |
| 8 | LIG1 |  |  | 691 | 0.171 | 0.0372 | No |
| 9 | BCAP31 |  |  | 737 | 0.166 | 0.0423 | No |
| 10 | RFC4 |  |  | 835 | 0.154 | 0.0407 | No |
| 11 | ADCY6 |  |  | 865 | 0.151 | 0.0468 | No |
| 12 | RNMT |  |  | 995 | 0.138 | 0.0405 | No |
| 13 | NT5C3A |  |  | 1263 | 0.117 | 0.0169 | No |
| 14 | PRIM1 |  |  | 1411 | 0.108 | 0.0066 | No |
| 15 | TAF1C |  |  | 1503 | 0.103 | 0.0025 | No |
| 16 | POM121 |  |  | 1692 | 0.092 | -0.0135 | No |
| 17 | SRSF6 |  |  | 2027 | 0.075 | -0.0476 | No |
| 18 | XPC |  |  | 2135 | 0.071 | -0.0555 | No |
| 19 | DDB1 |  |  | 2190 | 0.069 | -0.0575 | No |
| 20 | POLD3 |  |  | 2204 | 0.068 | -0.0547 | No |
| 21 | TMED2 |  |  | 2244 | 0.066 | -0.0551 | No |
| 22 | POLA1 |  |  | 2330 | 0.063 | -0.0610 | No |
| 23 | ERCC4 |  |  | 2421 | 0.060 | -0.0677 | No |
| 24 | NELFCD |  |  | 2426 | 0.060 | -0.0644 | No |
| 25 | PDE4B |  |  | 2443 | 0.059 | -0.0626 | No |
| 26 | BCAM |  |  | 2669 | 0.051 | -0.0855 | No |
| 27 | RFC3 |  |  | 2708 | 0.050 | -0.0868 | No |
| 28 | RAE1 |  |  | 2763 | 0.048 | -0.0900 | No |
| 29 | CANT1 |  |  | 2901 | 0.044 | -0.1031 | No |
| 30 | DCTN4 |  |  | 2906 | 0.044 | -0.1008 | No |
| 31 | GTF2B |  |  | 2948 | 0.042 | -0.1029 | No |
| 32 | ERCC2 |  |  | 3049 | 0.040 | -0.1120 | No |
| 33 | UPF3B |  |  | 3200 | 0.036 | -0.1272 | No |
| 34 | FEN1 |  |  | 3406 | 0.030 | -0.1490 | No |
| 35 | SF3A3 |  |  | 3425 | 0.030 | -0.1492 | No |
| 36 | GSDME |  |  | 3556 | 0.027 | -0.1627 | No |
| 37 | NT5C |  |  | 3633 | 0.025 | -0.1699 | No |
| 38 | GTF2F1 |  |  | 3765 | 0.021 | -0.1838 | No |
| 39 | NFX1 |  |  | 3784 | 0.020 | -0.1846 | No |
| 40 | USP11 |  |  | 3829 | 0.019 | -0.1885 | No |
| 41 | POLA2 |  |  | 3855 | 0.019 | -0.1902 | No |
| 42 | ELOA |  |  | 3871 | 0.018 | -0.1908 | No |
| 43 | SUPT5H |  |  | 3896 | 0.018 | -0.1925 | No |
| 44 | POLR1C |  |  | 3916 | 0.017 | -0.1936 | No |
| 45 | PCNA |  |  | 3986 | 0.016 | -0.2006 | No |
| 46 | SMAD5 |  |  | 4201 | 0.010 | -0.2248 | No |
| 47 | SNAPC4 |  |  | 4244 | 0.008 | -0.2292 | No |
| 48 | GMPR2 |  |  | 4248 | 0.008 | -0.2290 | No |
| 49 | UMPS |  |  | 4508 | 0.001 | -0.2590 | No |
| 50 | NME3 |  |  | 4830 | -0.007 | -0.2958 | No |
| 51 | ZWINT |  |  | 4930 | -0.009 | -0.3067 | No |
| 52 | ERCC3 |  |  | 4956 | -0.010 | -0.3089 | No |
| 53 | TK2 |  |  | 4975 | -0.010 | -0.3104 | No |
| 54 | RRM2B |  |  | 5030 | -0.012 | -0.3159 | No |
| 55 | RPA2 |  |  | 5078 | -0.013 | -0.3205 | No |
| 56 | NCBP2 |  |  | 5356 | -0.020 | -0.3514 | No |
| 57 | TARBP2 |  |  | 5534 | -0.025 | -0.3704 | No |
| 58 | CSTF3 |  |  | 5648 | -0.028 | -0.3817 | No |
| 59 | GTF3C5 |  |  | 5697 | -0.030 | -0.3854 | No |
| 60 | TP53 |  |  | 5700 | -0.030 | -0.3837 | No |
| 61 | AAAS |  |  | 5810 | -0.033 | -0.3943 | No |
| 62 | NUDT21 |  |  | 5874 | -0.035 | -0.3994 | No |
| 63 | POLR2H |  |  | 6016 | -0.040 | -0.4132 | No |
| 64 | TAF6 |  |  | 6120 | -0.044 | -0.4224 | No |
| 65 | RFC5 |  |  | 6216 | -0.047 | -0.4305 | No |
| 66 | GTF2H3 |  |  | 6414 | -0.053 | -0.4500 | No |
| 67 | GTF2H1 |  |  | 6499 | -0.056 | -0.4562 | No |
| 68 | POLB |  |  | 6591 | -0.060 | -0.4630 | No |
| 69 | VPS37B |  |  | 6650 | -0.064 | -0.4658 | No |
| 70 | SAC3D1 |  |  | 6692 | -0.065 | -0.4664 | No |
| 71 | DDB2 |  |  | 6772 | -0.070 | -0.4712 | No |
| 72 | NELFB |  |  | 7095 | -0.087 | -0.5031 | No |
| 73 | POLR2D |  |  | 7178 | -0.092 | -0.5068 | No |
| 74 | AK3 |  |  | 7253 | -0.097 | -0.5093 | No |
| 75 | POLR2C |  |  | 7313 | -0.102 | -0.5098 | Yes |
| 76 | IMPDH2 |  |  | 7333 | -0.103 | -0.5056 | Yes |
| 77 | VPS28 |  |  | 7378 | -0.107 | -0.5040 | Yes |
| 78 | EIF1B |  |  | 7425 | -0.110 | -0.5024 | Yes |
| 79 | MRPL40 |  |  | 7431 | -0.111 | -0.4961 | Yes |
| 80 | CETN2 |  |  | 7440 | -0.112 | -0.4900 | Yes |
| 81 | SDCBP |  |  | 7454 | -0.113 | -0.4845 | Yes |
| 82 | TAF13 |  |  | 7494 | -0.116 | -0.4817 | Yes |
| 83 | SSRP1 |  |  | 7499 | -0.117 | -0.4749 | Yes |
| 84 | ITPA |  |  | 7502 | -0.117 | -0.4678 | Yes |
| 85 | NELFE |  |  | 7517 | -0.119 | -0.4620 | Yes |
| 86 | RALA |  |  | 7581 | -0.125 | -0.4615 | Yes |
| 87 | DUT |  |  | 7608 | -0.128 | -0.4564 | Yes |
| 88 | POLR2G |  |  | 7656 | -0.133 | -0.4535 | Yes |
| 89 | HPRT1 |  |  | 7679 | -0.137 | -0.4475 | Yes |
| 90 | POLR3GL |  |  | 7702 | -0.140 | -0.4414 | Yes |
| 91 | POLR1D |  |  | 7706 | -0.140 | -0.4329 | Yes |
| 92 | RFC2 |  |  | 7739 | -0.144 | -0.4276 | Yes |
| 93 | TSG101 |  |  | 7759 | -0.147 | -0.4206 | Yes |
| 94 | MPC2 |  |  | 7783 | -0.149 | -0.4140 | Yes |
| 95 | TAF12 |  |  | 7788 | -0.150 | -0.4050 | Yes |
| 96 | TYMS |  |  | 7792 | -0.150 | -0.3960 | Yes |
| 97 | ERCC1 |  |  | 7810 | -0.154 | -0.3884 | Yes |
| 98 | NME1 |  |  | 7811 | -0.154 | -0.3787 | Yes |
| 99 | ALYREF |  |  | 7830 | -0.156 | -0.3711 | Yes |
| 100 | MPG |  |  | 7870 | -0.162 | -0.3655 | Yes |
| 101 | SURF1 |  |  | 7911 | -0.169 | -0.3596 | Yes |
| 102 | SNAPC5 |  |  | 7962 | -0.179 | -0.3542 | Yes |
| 103 | ADRM1 |  |  | 8001 | -0.186 | -0.3470 | Yes |
| 104 | RPA3 |  |  | 8014 | -0.188 | -0.3366 | Yes |
| 105 | TAF9 |  |  | 8142 | -0.214 | -0.3380 | Yes |
| 106 | POLE4 |  |  | 8169 | -0.220 | -0.3272 | Yes |
| 107 | EDF1 |  |  | 8210 | -0.231 | -0.3174 | Yes |
| 108 | GTF2H5 |  |  | 8243 | -0.238 | -0.3063 | Yes |
| 109 | DGUOK |  |  | 8252 | -0.242 | -0.2920 | Yes |
| 110 | GTF2A2 |  |  | 8261 | -0.243 | -0.2777 | Yes |
| 111 | POLR2F |  |  | 8262 | -0.244 | -0.2625 | Yes |
| 112 | SUPT4H1 |  |  | 8314 | -0.261 | -0.2521 | Yes |
| 113 | POLR2K |  |  | 8349 | -0.273 | -0.2390 | Yes |
| 114 | POLR2E |  |  | 8399 | -0.296 | -0.2262 | Yes |
| 115 | APRT |  |  | 8403 | -0.297 | -0.2079 | Yes |
| 116 | COX17 |  |  | 8453 | -0.316 | -0.1939 | Yes |
| 117 | RBX1 |  |  | 8469 | -0.324 | -0.1753 | Yes |
| 118 | NME4 |  |  | 8630 | -0.434 | -0.1667 | Yes |
| 119 | POLR2I |  |  | 8643 | -0.447 | -0.1401 | Yes |
| 120 | POLR2J |  |  | 8695 | -0.513 | -0.1140 | Yes |
| 121 | TAF10 |  |  | 8720 | -0.563 | -0.0816 | Yes |
| 122 | GUK1 |  |  | 8738 | -0.668 | -0.0418 | Yes |
| 123 | GPX4 |  |  | 8742 | -0.690 | 0.0010 | Yes |
Table: GSEA details [plain text format]

  

Fig 2: HALLMARK\_DNA\_REPAIR: Random ES distribution      
 Gene set null distribution of ES for **HALLMARK\_DNA\_REPAIR**
